# Case Report: Duodenal gastrointestinal stromal tumor misdiagnosed as tumor located on the major duodenal papilla leading to fatal gastrointestinal bleeding in a child

**DOI:** 10.3389/fped.2025.1546914

**Published:** 2025-03-07

**Authors:** Chengxian Yang, Kewei Li, Bo Xiang

**Affiliations:** Department of Pediatric Surgery, West China Hospital of Sichuan University, Chengdu, China

**Keywords:** child, duodenal gastrointestinal stromal tumor (dGIST), gastrointestinal hemorrhage or bleeding, fatal bleeding, misdiagnose analysis

## Abstract

**Background:**

Although gastrointestinal stromal tumors (GISTs) are the most common mesenchymal tumors of the gastrointestinal tract, they are rare in children, particularly those located on the duodenum. Here, we present an interesting pediatric case involving a 13-year-old boy who experienced gastrointestinal hemorrhage, he was misdiagnosed with a tumor located on the major duodenal papilla and was ultimately confirmed to be duodenal GISTs.

**Case presentation:**

A 13-year-old boy presented to a local hospital with fatigue and melena. Gastroscopy suggested a tumor located at the major duodenal papilla, and the patient was referred to our hospital for surgical evaluation. Upon further investigation and surgical exploration, the diagnosis was revised to a duodenal GIST with surface ulceration and active bleeding. The ulcer's morphology and location mimicked the appearance of the major duodenal papilla, leading to the initial diagnostic error.

**Conclusions:**

Duodenal GISTs in pediatric patients often present asymptomatically but can manifest with severe complications such as fatal gastrointestinal bleeding. The tumor's morphology and location can obscure the major papilla, complicating preoperative diagnosis and influencing surgical decision-making. Comprehensive preoperative evaluation and careful intraoperative exploration are critical for accurate diagnosis and optimal management.

## Background

Gastrointestinal stromal tumors (GISTs) are the most common mesenchymal tumors of the gastrointestinal tract, but they are rare in pediatric populations. The majority of cases (approximately 75%) occur in individuals over 50 years of age, with duodenal GISTs accounting for only 3%–5% of all GISTs cases (1, 2). Duodenal GISTs typically do not present with specific symptoms, manifestations such as hematemesis and gastrointestinal bleeding may arise depending on the tumor's location, size, and potential ulceration. This nonspecific presentation can lead to misdiagnosis, as duodenal GISTs may be confused with other lesions such as pancreatic pseudopapillary neoplasms or neuroendocrine tumors (3). Definitive diagnosis of duodenal GISTs relies on pathological examination, including immunohistochemical staining for markers such as CD117, CD34, and DOG1, which are characteristic of these tumors (4).

Treatment strategies for duodenal GISTs remain a subject of debate. Pancreaticoduodenectomy (PD) and local resection (LR) are the primary surgical options, with the choice largely influenced by the tumor's size, location, and growth pattern (5). PD is generally favored for tumors near the ampulla of Vater or those with extensive local invasion, while LR may be appropriate for smaller, well-localized tumors with low-risk features (6). Further research and long-term outcomes studies are needed to refine surgical guidelines and optimize patient management.

## Case presentation

**Figure 1:**
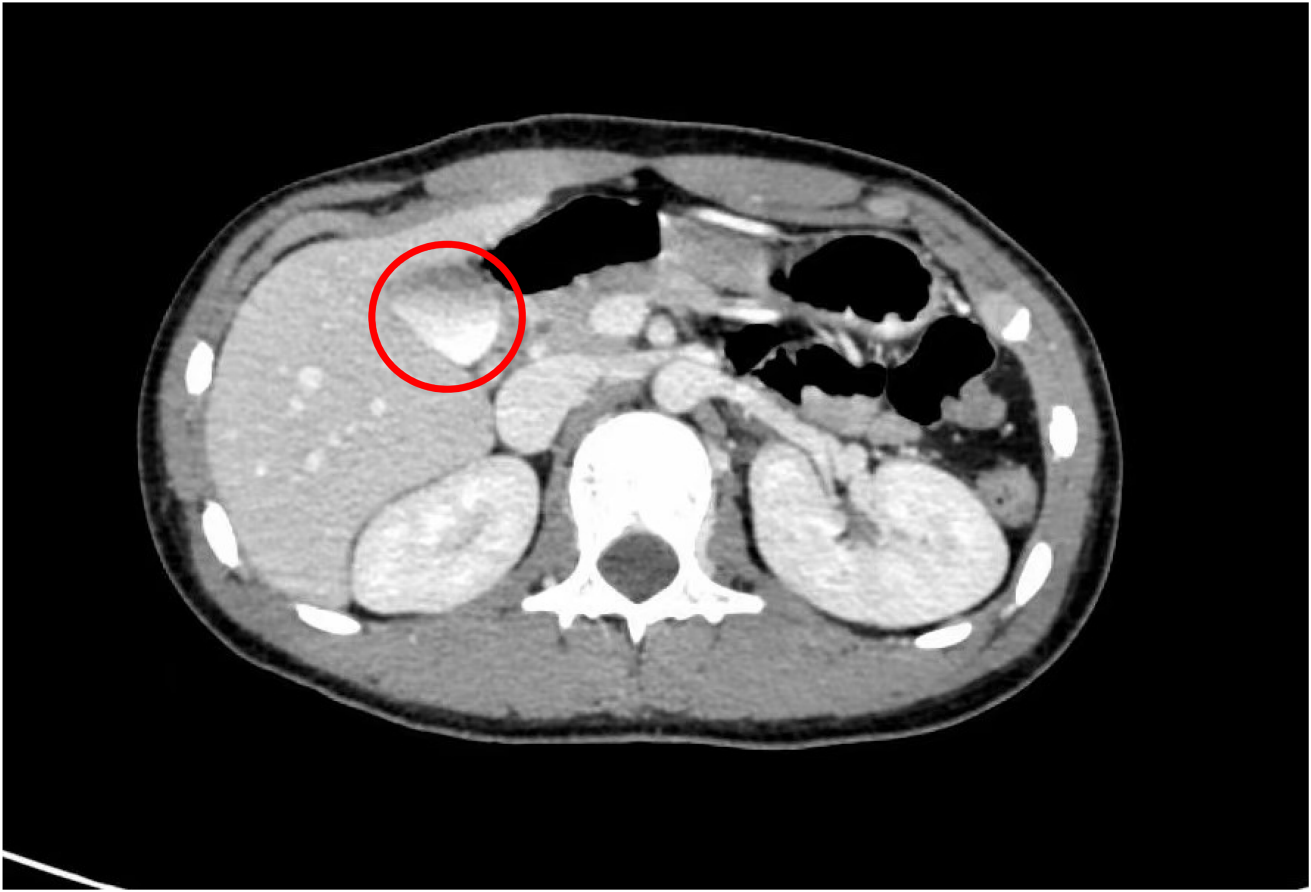
Preoperative CT scan, the tumor is located near the major duodenal papilla and is closely related to the pancreas (red circle).

The patient was a 13-year-old male who initially presented to a primary hospital with unexplained fatigue and melena. He reported the passage of approximately 200 ml of dark red stool without an apparent trigger, accompanied by palpitations and fatigue, but denied nausea, vomiting, hematemesis, diarrhea, or other symptoms. Suspecting upper gastrointestinal bleeding, the local hospital performed an esophagogastroduodenoscopy (EGD), which identified a firm mass located at the major duodenal papilla, encasing the ampulla. No definite bleeding point was observed under endoscopy, except for a visible “opening” on the tumor surface, which was presumed to be the duodenal papillary orifice based on its location. The patient was subsequently referred to our hospital for further evaluation and surgical planning. Contrast-enhanced computed tomography (CT) performed at our institution revealed a mixed-density soft tissue nodule in the descending duodenum, measuring approximately 2.3 × 2.2 cm, with close anatomical association with the pancreatic head and inferior vena cava (Figure 1).

Upon admission to our hospital, the patient's vital signs were closely monitored, and his condition initially stabilized. His temperature, blood pressure, and heart rate were within normal ranges. However, within 10 h of admission, his hemoglobin level decreased from 80 g/L to 68 g/L, accompanied by tachycardia and further pallor of the skin and lips, suggesting ongoing active bleeding. Therefore, with the consent of the patient's family, we proceeded with an emergency exploratory laparotomy to identify the bleeding source, control hemorrhage, and address the underlying disease.

**Figure 2:**
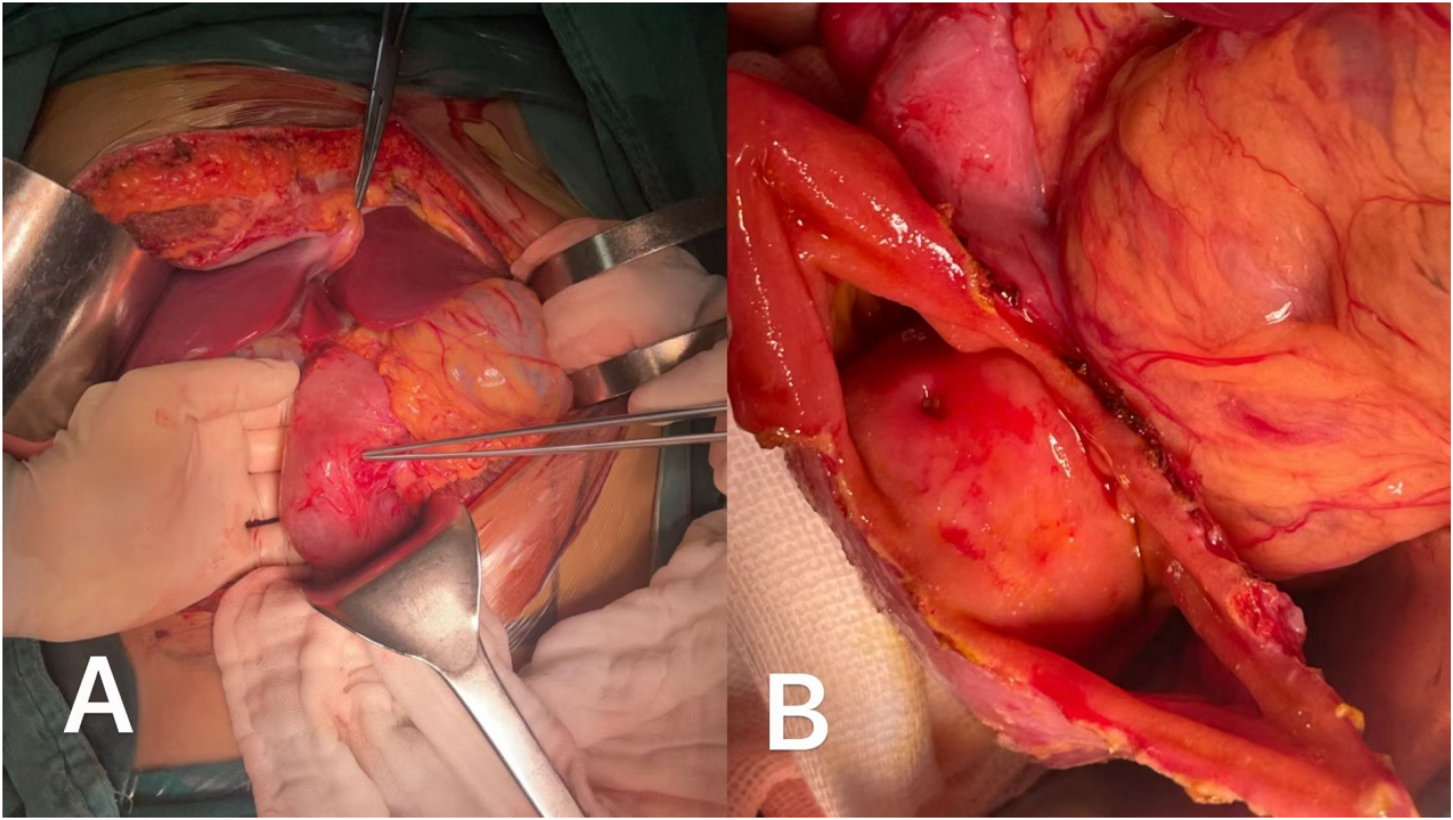
**(A)** The tumor originated from mesangial margin of the intestinal mucosa and grew endogeneously. **(B)** The ulcer on the surface of the tumor appeared indistinguishable from the opening of the major duodenal papilla.

Intraoperatively, a broad-based, pliable tumor originating from the mesangial margin of the duodenal mucosa was identified in the descending duodenum (Figure 2A). The tumor measured approximately 3 cm × 4 cm and was highly vascularized. Surface erythema and ulceration were observed, with active bleeding at the ulcerated site (Figure 2B). Notably, the major duodenal papilla orifice was located approximately 1.5 cm superior to the tumor, and bile and pancreatic fluid were observed to flow freely from this orifice upon gallbladder compression (Supplementary Video S1). No significant abnormalities were noted in the remaining duodenum or pancreatic head. Exploration of the abdominal cavity revealed no other remarkable findings, and no blood accumulation was detected.

Based on these findings, we performed a local resection (LR) of the tumor. After full exposure of the tumor, it was completely excised along with a portion of the duodenal wall, and the duodenal incision was closed with double-layered inverted sutures. Postoperative examination of the tumor revealed fish-meat-like necrosis within the tumor mass, and the ulceration extended to the deep portions of the tumor with surrounding tissue necrosis.

**Figure 3:**
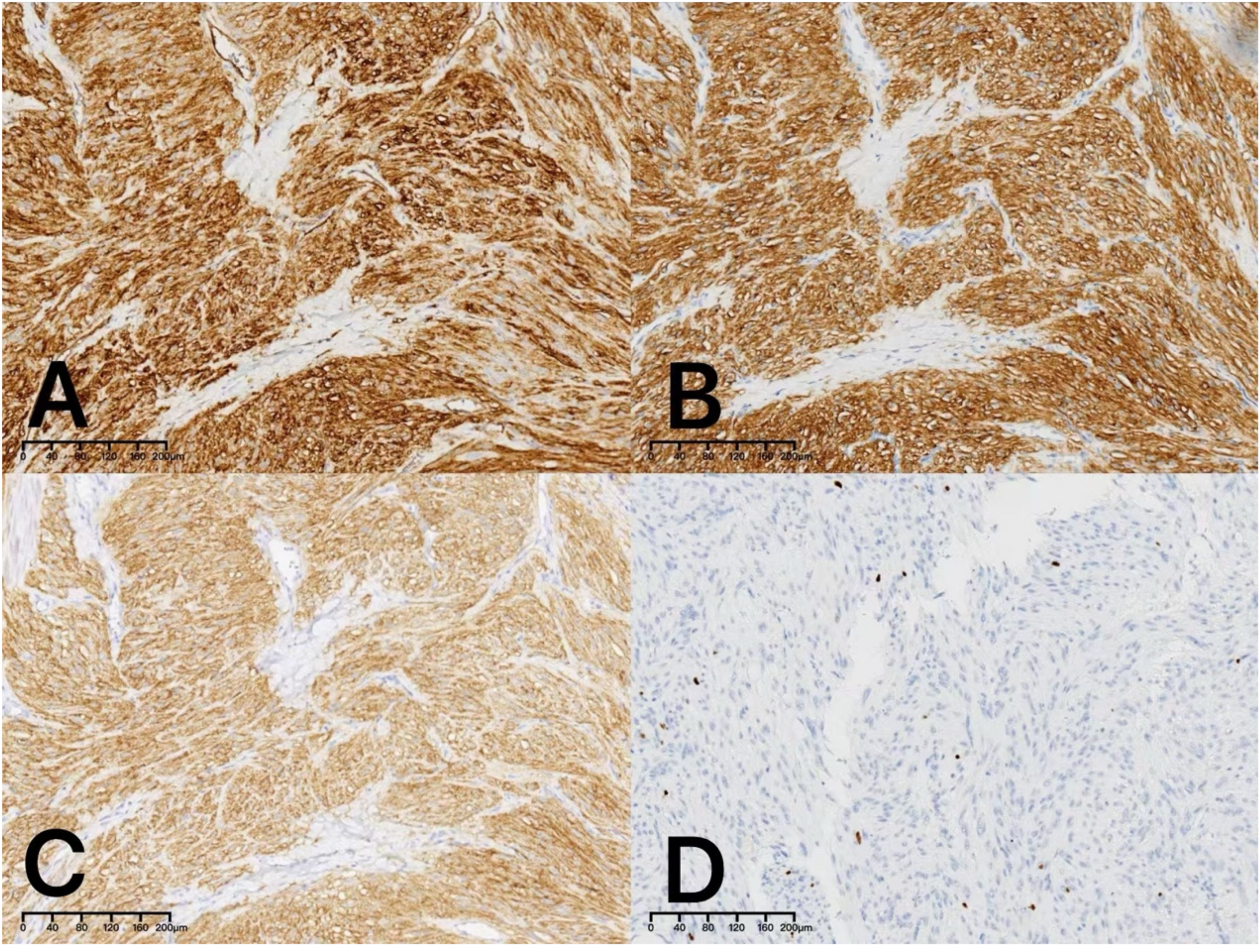
Immunohistochemical examination revealed positive staining for CD34 **(A:**×10**)**, CD117 (**B:**×10**)** and DOG1 **(C:**×10**)**. The Ki-67 proliferation index was approximately 2% **(D:**×10**)**.

Postoperative immunohistochemistry analysis confirmed the diagnosis of a low-risk gastrointestinal stromal tumor. The tumor exhibited strong positivity for CD117 (Figure 3A), CD34 (Figure 3B), and DOG1 (Figure 3C); negativity for SMA. The Ki-67 proliferation index was approximately 2% (Figure 3D), and mitotic counts ranged from 0 to 2 per 5 square millimeters, consistent with a low malignant potential.

**Figure 4:**
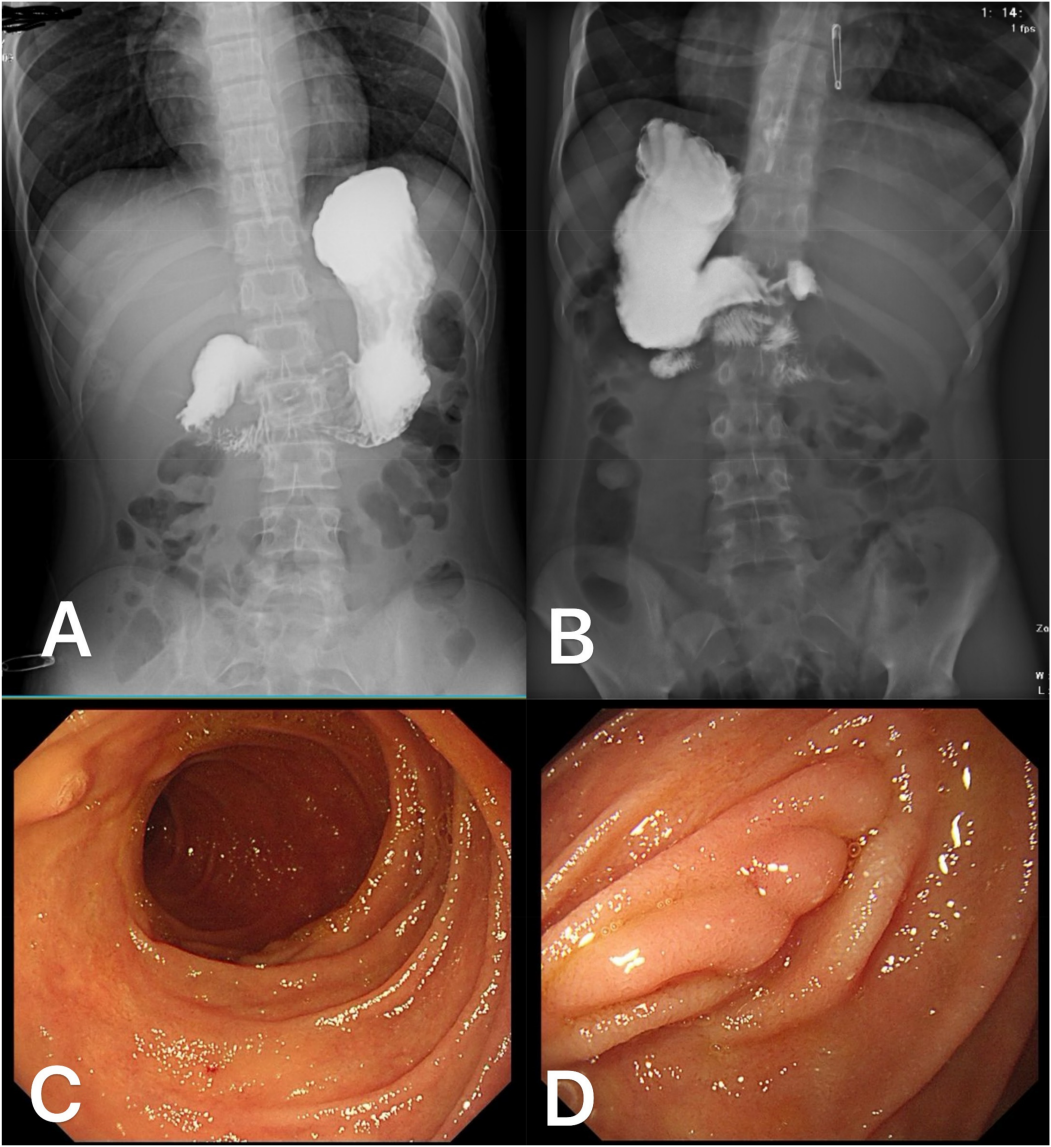
Postoperative follow-up data of the patient: One month after surgery, an upper gastrointestinal barium contrast study in the anteroposterior **(A)** and posterior **(B)** views demonstrated smooth passage of the contrast agent without signs of obstruction or leakage, with good duodenal peristalsis. At six months postoperatively, esophagogastroduodenoscopy revealed smooth duodenal mucosa without evidence of ulceration or tumor **(C)** The mucosal folds in the surgical area of the descending duodenum appeared nodular **(D)**.

Postoperatively, the patient received gastrointestinal decompression, infection prophylaxis, parenteral nutrition support, proton pump inhibitors (PPIs) to suppress gastric acid secretion, and gastric mucosal protection therapy. Close monitoring of the patient's overall condition, vital signs, and drainage status was conducted. Oral intake was gradually reintroduced following the recovery of gastrointestinal function, and the postoperative recovery process was uneventful. Approximately one month after surgery, an upper gastrointestinal barium contrast study was performed (Figures 4A,B), which demonstrated smooth passage of the contrast agent without evidence of obstruction or leakage, and good duodenal peristalsis. During mid- to long-term follow-up, an esophagogastroduodenoscopy was conducted six months postoperatively (Figures 4C,D), revealing smooth duodenal mucosa with no signs of ulceration or neoplasia. Postoperative changes were observed in the descending duodenum, characterized by mucosal folds presenting as small nodular formations. Additionally, follow-up examinations showed no signs of tumor recurrence.

Based on the patient's postoperative pathological findings and the established risk stratification criteria for GISTs, the patient was classified as low-risk. Consequently, adjuvant imatinib therapy was not administered.

## Discussion and conclusion

Gastrointestinal stromal tumors (GISTs) are extremely rare in the pediatric population, accounting for only 0.5%–2.7% of all GIST cases. Among these, duodenal GISTs are even rarer. Pediatric GISTs differ significantly from adult GISTs in clinical presentation, molecular characteristics, and treatment response. A review of the literature shows that most pediatric GISTs are wild-type GISTs, lacking KIT/PDGFRA mutations (7). Many pediatric GISTs are associated with alterations in the succinate dehydrogenase (SDH) complex, particularly SDH-deficient GISTs, and are more likely to occur in the stomach (8), which represents a significant proportion of pediatric cases. Due to these molecular features, pediatric GISTs exhibit poor responses to conventional tyrosine kinase inhibitors (TKIs) such as imatinib. Consequently, the treatment strategy primarily advocates surgical resection, with the decision for adjuvant therapy postoperatively based on risk stratification and molecular phenotype assessment.

Upon reviewing prior cases, we identified reports of pediatric duodenal GISTs, including cases involving a 14-year-old and a 7-year-old boy (9, 10). Notably, both presented with acute gastrointestinal bleeding and had negative preoperative endoscopic results. Both ultimately required emergency exploratory surgery, which confirmed the diagnosis of GIST. Clinically, they presented with long-standing anemia or gastrointestinal symptoms such as vomiting and melena. In contrast, the case we report involves a 13-year-old male who also presented with acute gastrointestinal bleeding. Preoperative misdiagnosis suggested a tumor involving the ampulla, but further investigation revealed that the lesion was a duodenal GIST with surface ulceration and active bleeding. Unlike the previously reported cases, our patient did not have long-standing chronic symptoms, and the lesion was identified during preoperative gastroduodenoscopy. Due to its anatomical location, it was initially misinterpreted as a lesion involving the ampulla, and the ulcer on the tumor's surface was mistaken for the opening of the major duodenal papilla. This led to an initial suspicion of pancreatic head involvement and a potential need for pancreaticoduodenectomy (PD). This case highlights the diagnostic challenges posed by the tumor's anatomical location and morphology. This diagnostic error emphasizes the difficulties in endoscopic evaluation of duodenal lesions, particularly when ulcerative changes obscure critical anatomical landmarks (6, 11). The case underscores the importance of comprehensive preoperative evaluation and careful intraoperative exploration in ensuring accurate diagnosis and optimizing treatment.

For this patient, given the deterioration of his condition, characterized by a significant drop in hemoglobin and signs of active gastrointestinal bleeding, an emergency exploratory laparotomy was performed. Intraoperatively, the tumor was found to be broad-based and pliable, originating from the intestinal mucosa without involvement of the ampulla. Despite the tumor's location and appearance on endoscopy resembling that of an ampullary lesion, intraoperative exploration clarified its relationship with surrounding structures, making LR a feasible option. After tumor removal, the actual duodenal papilla was exposed, and smooth bile discharge was observed when the gallbladder was compressed.

The choice between PD and LR remains controversial due to the significant trauma associated with PD surgery and its profound impact on prognosis in pediatric patients. Based on previous studies, this decision depends on multiple factors, including tumor size, location, involvement of adjacent structures, and the experience of the surgical team. PD, also known as the Whipple procedure, involves *en bloc* resection of the duodenum, pancreatic head, bile duct, and surrounding structures. It is primarily indicated for large tumors located near the ampulla or those suspected of invading the pancreas or bile ducts. PD increases the likelihood of achieving R0 resection, which is critical for reducing the risk of recurrence in high-risk GISTs (5). However, this procedure is complex and associated with a higher incidence of complications, including pancreatic fistula, delayed gastric emptying, and biliary leakage (12). In contrast, LR involves excising the tumor with minimal disruption to surrounding structures, preserving pancreatic and biliary function, and is particularly suitable for small, well-demarcated tumors located away from the ampulla. LR is associated with fewer complications, shorter operative times, and faster recovery compared to PD (5). However, for tumors with poorly defined margins or those near the ampulla, achieving R0 resection may be difficult, increasing the risk of local recurrence (6). The decision to perform PD vs. LR should be guided by the following factors:

(1) **Tumor location**: Tumors near the ampulla or involving the pancreatic head are better managed with PD to ensure R0 resection, whereas tumors distant from the ampulla are more suitable for LR.
(2) **Tumor size and risk profile**: Large, high-risk GISTs with aggressive features may warrant PD, while small, low-risk tumors are better suited for LR (13).
(3) **Patient factors**: Patients with severe comorbidities or those unable to tolerate major surgery are better candidates for LR.
(4) **Surgical expertise and resources**: Performing PD requires an experienced surgical team and appropriate institutional resources, which is a critical consideration in decision-making.

In this case, LR was selected due to the tumor's small size, absence of ampullary involvement, and the goal of minimizing surgical trauma (12). Based on current research, adjuvant therapy is recommended to begin 4–8 weeks postoperatively. Reichardt et al. (14) highlighted the importance of tailoring adjuvant therapy based on recurrence risk in their review of GIST adjuvant treatment. For low-risk patients, adjuvant therapy should be considered with caution. The postoperative pathological analysis in this case confirmed the tumor as a low-risk GIST, and therefore no adjuvant therapy was administered. Postoperatively, the patient was monitored with imaging assessments every 3–6 months, with no evidence of recurrence or metastasis, underscoring the importance of individualized treatment planning in managing duodenal GISTs.

This case highlights the critical importance of precise preoperative evaluation and reminds clinicians to remain vigilant in recognizing rare causes of gastrointestinal bleeding in pediatric patients. Endoscopic findings should be corroborated with imaging studies and intraoperative exploration to ensure accurate diagnosis and formulate a rational surgical strategy. The choice of surgical approach for duodenal GISTs is crucial, particularly in pediatric patients, and should weigh multiple factors to optimize therapeutic outcomes.

## Data Availability

The original contributions presented in the study are included in the article/Supplementary Material, further inquiries can be directed to the corresponding authors.
